# Transfection of Sertoli cells with androgen receptor alters gene expression without androgen stimulation

**DOI:** 10.1186/s12867-015-0051-7

**Published:** 2015-12-29

**Authors:** D. Fietz, M. Markmann, D. Lang, L. Konrad, J. Geyer, S. Kliesch, T. Chakraborty, H. Hossain, M. Bergmann

**Affiliations:** Institute of Veterinary Anatomy, Histology and Embryology, Justus Liebig University, Frankfurter Straße 98, 35392 Giessen, Germany; Institute of Medical Microbiology, Justus Liebig University, Giessen, Germany; Department of Gynecology and Obstetrics, Justus Liebig University, Giessen, Germany; Department of Clinical Andrology, Centre for Reproductive Medicine and Andrology, University Clinic Münster, Münster, Germany

**Keywords:** Transfection, Gene expression analysis, Androgen receptor, Sertoli cells

## Abstract

**Background:**

Androgens play an important role for the development of male fertility and gained interest as growth and survival factors for certain types of cancer. Androgens act via the androgen receptor (AR/*Ar*), which is involved in various cell biological processes such as sex differentiation. To study the functional mechanisms of androgen action, cell culture systems and AR-transfected cell lines are needed. Transfection of AR into cell lines and subsequent gene expression analysis after androgen treatment is well established to investigate the molecular biology of target cells. However, it remains unclear how the transfection with AR itself can modulate the gene expression even without androgen stimulation. Therefore, we transfected *Ar*-deficient rat Sertoli cells 93RS2 by electroporation using a full length human AR.

**Results:**

Transfection success was confirmed by Western Blotting, immunofluorescence and RT-PCR. AR transfection-related gene expression alterations were detected with microarray-based genome-wide expression profiling of transfected and non-transfected 93RS2 cells without androgen stimulation. Microarray analysis revealed 672 differentially regulated genes with 200 up- and 472 down-regulated genes. These genes could be assigned to four major biological categories (development, hormone response, immune response and metabolism). Microarray results were confirmed by quantitative RT-PCR analysis for 22 candidate genes.

**Conclusion:**

We conclude from our data, that the transfection of *Ar*-deficient Sertoli cells with AR has a measurable effect on gene expression even without androgen stimulation and cause Sertoli cell damage. Studies using AR-transfected cells, subsequently stimulated, should consider alterations in AR-dependent gene expression as off-target effects of the AR transfection itself.

**Electronic supplementary material:**

The online version of this article (doi:10.1186/s12867-015-0051-7) contains supplementary material, which is available to authorized users.

## Background

Androgens play a pivotal role for the development of the male phenotype, the initiation and maintenance of spermatogenesis and therefore male fertility (for review see [1]). The action of the most important androgens testosterone (T) and dihydrotestosterone (DHT) is mediated by the androgen receptor (AR/*Ar*). It is a ligand-activated transcriptional factor belonging to the nuclear receptor superfamily. The AR/*Ar* gene is located on the X chromosome and consists of eight exons, coding for the N-terminal transcription regulation domain, the DNA binding domain (DBD) in the middle of the protein and the C-terminal ligand binding domain (LBD). The DBD as well as the LBD are highly conserved throughout species (for review see [2]). Bound to its ligand, the androgen-AR complex is translocated into the nucleus, binds to the DNA (androgen responsive elements, AREs) and is able to activate or repress gene expression by recruiting co-activators or co-repressors (for review see [3]). The activity of steroid hormone receptors is also regulated by post-transcriptional modifications. In case of AR/*Ar*, a great variety of these modifications has been described, i.e. phosphorylation, acetylation, ubiquitination and also methylation (for review see [4]).

The AR/*Ar* is expressed in all tissues except the spleen (for review see [2]). In the testis, it is expressed in interstitial Leydig cells and endothelial cells, as well as in peritubular myoid cells and tubular Sertoli cells [5], for review see [1]. Since germ cells do not express AR/*Ar*, the androgen action has to be mediated towards the germ cells by Sertoli cells. These somatic cells have been described as branched cells surrounding all germ cell stages [6, 7]. As was shown by Willems et al. [8], a selective ablation of *Ar* in mouse Sertoli cells (SCARKO) leads to a disturbed Sertoli cell maturation including a delayed and defective establishment of the blood-testis barrier. Moreover, no meiotic germ cells were observed in SCARKO mice, showing the importance of a functional AR/*Ar* on Sertoli cell biology and for the development of germ cells.

To examine the role of the AR/*Ar* in different biological processes such as cell growth and survival as well as AR/*Ar*-dependent gene expression, cell culture systems are needed. Therefore, administration of T and/or the more efficient metabolite DHT has widely been used to investigate the effect of androgens and AR/*Ar*, respectively, in diverse cultured cells such as human breast cancer cells, adrenocortical carcinoma cells, murine skeletal muscle cells or liver carcinoma cells [9–12]. Additionally, AR/*Ar*-deficient cell lines were used, e.g. AR-deficient MCF-7 breast cancer cells, to examine the effect on estrogen administration in a system lacking AR [13]. Szelei et al. [14] transfected AR-deficient MCF-7 breast cancer cells with human AR and showed an inhibition of proliferation. Also prostate cancer cells devoid of AR were transfected with human AR and showed a decreased proliferation rate [15]. The question is, whether the transfection procedure itself might have led to an altered expression of AR/*Ar*-dependent and AR/*Ar*-independent genes. Xiao et al. [16] demonstrated equal concerns in *Amh*-Cre-transfected mouse, where an increase of oxidative stress and lipid peroxidation in Sertoli cells was detected even without stimulation.

Beside “classical” androgen-dependent prostate cancer development, also androgen-independent signalling pathways gained increasing interest as shown recently by Li et al. [17]. The authors describe a persistent transcriptional activity in castration-resistant prostate cancer cell lines in the absence of androgens. This transcriptional activity was mediated by a truncated AR protein lacking the LBD. This raises the question, whether an androgen-independent AR/*Ar* action is always important in cell biology and which genes might be expressed or repressed by AR/*Ar* presence alone.

For this purpose, we transfected rat Sertoli cells which have been shown to be deficient of *Ar* with full length human AR DNA. After transfection, we performed genome-wide microarray analysis and compared the gene expression pattern with non-transfected Sertoli cells to identify a possible “intrinsic” activity of AR/*Ar* without androgen administration. We found significantly altered gene expression in transfected compared with non-transfected cells, possibly influencing Sertoli cell function.

## Results

### Transfection of 93RS2 cells with the human AR

**Fig. 1:**
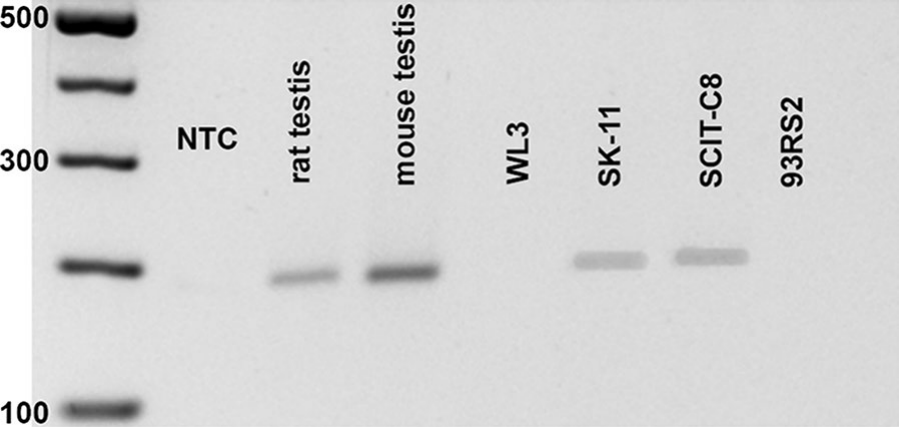
Expression of androgen receptor (*Ar*) mRNA in Sertoli cell cultures. To find an appropriate cell culture system for our planned transfection studies, RT-PCR with specific primers for mouse and rat *Ar* was performed. Testis homogenate from rat and mouse served as positive control, whereas water was used as no template control (NTC) samples. We tested two mouse (WL3 and SK-11) as well as two rat Sertoli cell lines (SCIT-C8 and 93RS2). The latter revealed no expression of intrinsic *Ar* and were therefore used for further experiments

Performing RT-PCR with primers specific for mouse and rat *Ar*, respectively, rat Sertoli cells (93RS2, [18]) proved to lack endogenous *Ar* (Fig. 1) and were therefore chosen for further experiments.

**Fig. 2:**
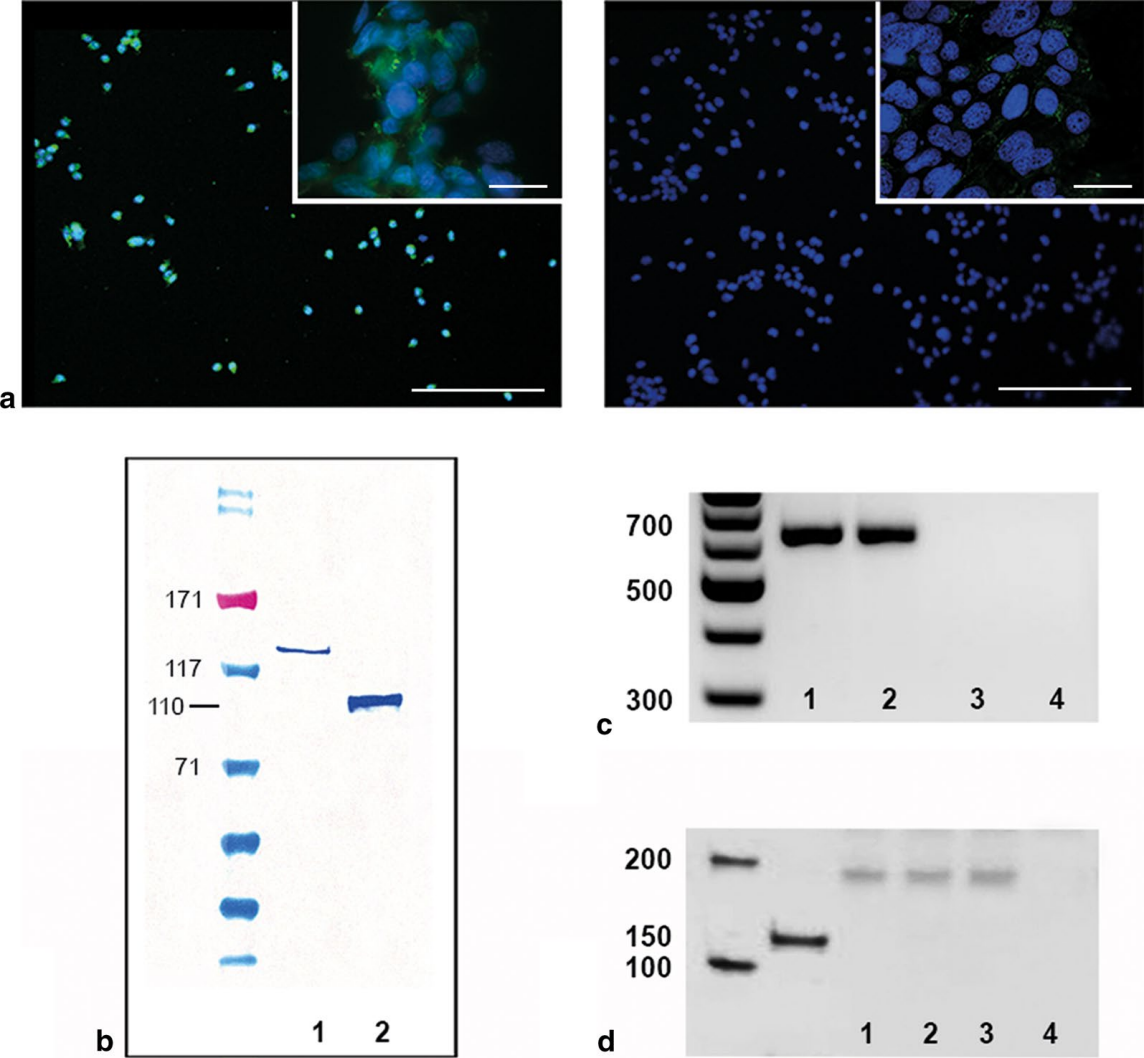
Transfection control of 93RS2 Sertoli cells. **a** 24 h after transfection, transfected (**a**) and non-transfected (**b**) cells as negative control were fixed for IF experiments. *left* Incubation with rabbit anti-GFP antibody showed successful transfection of almost 80 % of cells*. right* No staining signal was detectable in non-transfected cells. *Scale bars* in main image: 200 µm, detail: 25 µm. DAPI counterstain. **b** Western Blot analysis revealed AR protein in transfected Sertoli cells at approx. 135 kDa (*1*) and in human testis tissue at the expected molecular weight of 110 kDa (*2*). The higher protein weight measured in transfected cells is due to coupling of AR with GFP. **c** Expression of human AR mRNA was tested in human testis homogenate (*1*), transfected (*2*) and non-transfected cells (*3*). AR mRNA was detected in the positive control and transfected 93RS2hAR17 cells, but not in non-transfected cells and the NTC (*lane 4*). **d** To control the CAG repeat length in transfected 93RS2 cells, we performed high-resolution PAGE. Three different passages of 93RShAR17 cells (*lanes 1–3*) were analysed and revealed a band for human AR at 185 bp by using two different DNA ladders. By sequencing, 185 bp was shown to be typical for the presence of 17 CAG repeats. *Lane 4* no template control (NTC)

Success of transfection with full length human AR CDS was validated by immunofluorescence (IF, Fig. 2a), Western Blot (Fig. 2b) and RT-PCR (Fig. 2c). As the commercially available human AR was introduced in a GFP-coupled vector system, we used a rabbit anti-GFP antibody for IF experiments in transfected cells whereas non-transfected cells were used as internal negative control. Using PAGE, we were able to show the CAG repeat length of 17 to be stable throughout different settings (Fig. 2d).

### Microarray analysis revealed an altered gene expression in transfected 93RS2 cells

Microarray analysis revealed 672 significantly regulated genes (p < 0.01 and fold change (FC) >2.0). Of these, 200 genes showed higher gene expression values, whereas 472 revealed a lower gene expression in 93RShAR17 cells compared with non-transfected cells.

**Fig. 3:**
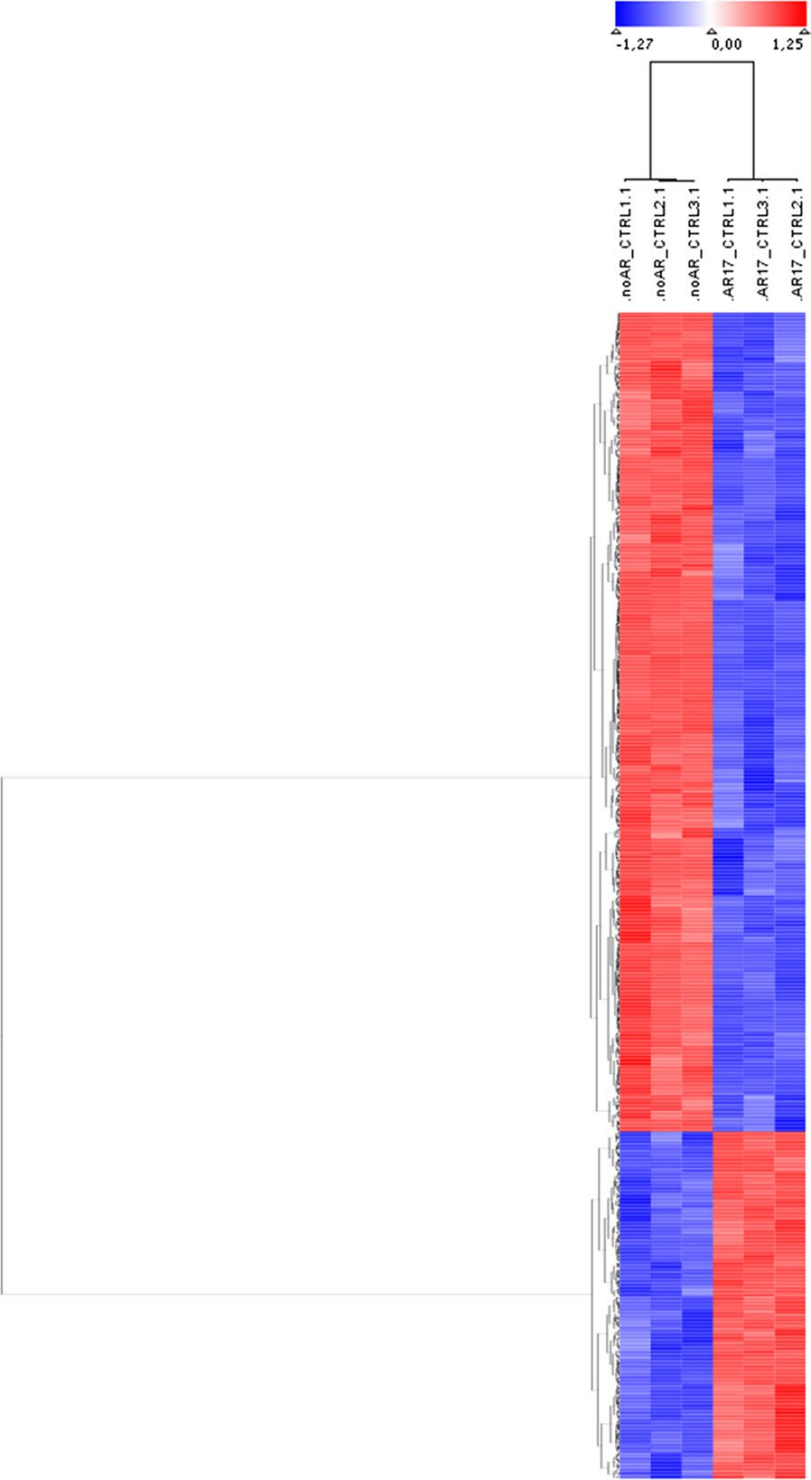
Hierarchical clustering of 672 significantly altered genes. *Genes* are depicted in *rows* and samples in *columns*. *Blue* indicates downregulation whereas red shows upregulation. Clustering was done using “Pearson correlation” and “complete linkage”. The tree on the* left* reflects the distances between gene profiles based on this algorithm

Hierarchical clustering of the 672 significantly regulated genes shows two clusters clearly differentiating between transfected and non-transfected cells (Fig. 3). Three biological replicates have been tested and show a homogeneous expression pattern, indicating high reproducibility of microarray results. An overview of the ten highest regulated genes for down- and up-regulation is given in Table 1. Complete array data may be found following the link provided [19].

**Table 1:** Overview of ten highest ranked up- and down-regulated genes

Regulation	Identifier	Symbol	EntrezID	FDR	FC	Gene name	Comment
Down	Idx_R307_C32	*Cybrd1*	295,669	0.001	−107,712	Cytochrome b reductase 1	Expression of the ferric reductase is regulated by intracellular iron concentration and other facilitators of iron absorption, indicating that it responds to iron demand
Down	Idx_R293_C42	*Tmsbl1*	286,978	0.003	−71,936	Thymosin beta-like protein 1	Actin cytoskeleton organization
Down	Idx_R29_C52	*Nnat*	94,270	0.001	−50,214	Neuronatin	The effects of Nnat on inflammatory pathways in vitro and in vivo suggest a pathophysiological role of this new gene in diabetic vascular diseases
Down	Idx_R245_C71	*Fam46a*	300,870	0.006	−42,921	Family with sequence similarity 46, member A	
Down	Idx_R259_C49	*Ctsz*	252,929	0.003	−39,163	Cathepsin Z	Accounts for the lysosome’s capacity to digest polyQ sequences. Cathepsins L and Z are important in defending against the accumulation and toxicity of polyQ proteins
Down	Idx_R322_C43	*Slc24a3*	85,267	0.003	−38,220	Solute carrier family 24 (sodium/potassium/calcium exchanger), member 3	
Down	Idx_R196_C66	*Nudt7*	361,413	0.004	−36,487	Nudix (nucleoside diphosphate linked moiety X)-type motif 7	
Down	Idx_R200_C18	*Marveld1*	309,375	0.001	−34,855	MARVEL domain containing 1	
Down	Idx_R240_C21	*Tpp1*	83,534	0.001	−32,489	Tripeptidyl peptidase I	
Down	Idx_R245_C74	*Tpp1*	83,534	0.001	−31,626	Tripeptidyl peptidase I	
Down	Idx_R47_C36	*Bhlhb9*	317,407	0.001	−30,042	Basic helix-loop-helix domain containing, class B, 9	
Up	Idx_R117_C7	*Irf7*	293,624	0.003	5540	Interferon regulatory factor 7	The crucial regulator of type I interferons (IFNs) against pathogenic infections, which activate IRF7 by triggering signaling cascades from pathogen recognition receptors (PRRs) that recognize pathogenic nucleic acids
Up	Idx_R14_C99	*Apol9a*	503,164	0.003	5611	Apolipoprotein L 9a	
Up	Idx_R252_C110	*Usp18*	312,688	0.003	5976	Ubiquitin specific peptidase 18	
Up	Idx_R317_C53	*Usp18*	312,688	0.003	6264	Ubiquitin specific peptidase 18	
Up	Idx_R74_C32	*Wfdc18*	171,059	0.004	6291	WAP four-disulfide core domain 18	
Up	Idx_R53_C102	*Ripk4*	304,053	0.001	6479	Receptor-interacting serine-threonine kinase 4	
Up	Idx_R278_C80	*Ccl4*	116,637	0.006	7177	Chemokine (C–C motif) ligand 4	
Up	Idx_R188_C91	*Oas1b*	246,268	0.003	7827	2–5 oligoadenylate synthetase 1B	
Up	Idx_R192_C96	*Ffar4*	294,075	0.004	9720	Free fatty acid receptor 4	
Up	Idx_R299_C11	*Il33*	361,749	0.002	9759	Interleukin 33	IL-33 is a dual function protein that may function as a proinflammatory cytokine and an intracellular nuclear factor with transcriptional regulatory properties
Up	Idx_R66_C107	*Il33*	361,749	0.001	10,690	Interleukin 33	
Up	Idx_R102_C39	*Mx1*	24,575	0.004	12,708	Myxovirus (influenza virus) resistance 1	The human myxovirus resistance protein 1 is a key mediator of the interferon-induced antiviral response against a wide range of viruses. MxA may form oligomeric rings around tubular nucleocapsid structures. As a consequence, these viral components are trapped and sorted to locations where they become unavailable for the generation of new virus particles

### “Development”, “Hormone response” and “Immune response” are the predominant functions of the differently regulated genes

Of 370 annotated down-regulated genes, 330 could be assigned to DAVID functional categories, and 124 out of 142 annotated up-regulated genes, respectively.

An overview of the functional categories that have been inferred with DAVID is given in Table 2. Down-regulation is predominant in “Cell development/Cell contact”, “Response to hormone stimulus” and “Nucleotide catabolic process”, whereas regulation is evenly distributed in “Immune response”. The highest score values are achieved by four significantly overrepresented gene ontology (GO) categories clustered under “Biological adhesion”, whereas the highest number of genes is assigned to 15 GO categories grouped as a cluster named “Epithelium development”. More than half of the functional assigned groups belong to cell development and cell contact while 25 % of the functionally assigned genes are related to immune response. 36 genes can be attributed to “Hormone stimulus” and a minority of 12 genes contributes to “Nucleotide catabolic process”.

**Table 2:** Overview of functional gene ontology categories according to their pattern of significantly regulated genes

Group	Cluster#	Cluster of GO categories	Score		Symbols
Cell development/cell contact [106]	1	Biological adhesion (4) [25]	2.27	Up	*Vnn1, Amigo2, Bcam, Cdh2, Ceacam1, Col12a1, Col14a1, Col16a1, Dsg2, Gpc1, Mcam, Omd, Sned1, Col18a1, Ctgf, Gpr56, Ncam1, Igfbp7*
				Down	*Itgb8, F5, Pcdh1, Pcdh18, Plcxd2, Ptprm, Ctgf*
	3	Axonogenesis (13) [36]	1.89	Up	*Aldh1a2, Apbb1, Apoe, Boc, Cd24, Cdkn1c, Chn2, Col18a1, Col18a1, Cxcl12, Efna2, Efnb1, Fgfr2, Gli2, Gpc2, H19, Hoxc10, Krt19, Lpar3, Nnat, Nrep, Obsl1, Pmp22, Ppp1r9a, Prickle2, Sdc2, Sema4f, Shroom3, Sox5, Uchl1*
				Down	*Ptprm, Epha7, Dpysl3, Mtss1, Nes, Sgk1*
	4	Retinoid metabolic process (5) [8]	1.75	Up	*Akr7a3, Aldh1a2, As3mt, Ldhb, Rarres2, Rbp1*
				Down	*Crabp2, Rbp2*
	7	Epithelium development (15) [50]	1.47	Up	*Acp5, Adamts1, Adck3, Aldh1a2, Celsr1, Col18a1, Col1a1, Col4a1, Cxcl12, Disp1, Efna2, Efnb1, Fbn1, Fgfr2, Foxe1, Foxl2, Gli2, H19, Hmx2, Hoxc10, Irf6, Kazn, Mgp, Mn1, Mycn, Pgf, Plce1, Serpinf1, Sfrp2, Shroom3, Sox5, Spry1, Srgn, Tbx18, Tbx4, Tek, Tgfb1i1, Tgm2, Upk1b*
				Down	*Ctgf, Crabp2, Fst, Ptger2, Rsad2, Cdx2, Hoxb6, Krt14, Ptgs2, Foxp2, Myc*
H [36]	2	Response to steroid hormone stimulus (10) [36]	1.90	Up	*Acp5, Adamts1, Adck3, Aldh1a2, Apoe, Boc, Cd24, Celsr1, Col1a1, Cxcl12, Disp1, Efna2, Efnb1, Fgfr2, Gli2, Gpr56, H19, Igfbp7, Krt19, Lpar3, Mgp, Ncam1, Nnat, Pgf, Plce1, Sdc2, Serpinf1, Sfrp2, Tek, Tgfb1i1, Tgm2*
				Down	*Foxp2, Myc, Nes, Ptgs2, Sgk1*
Immune response [55]	5	Innate immune response (4) [34]	1.69	Up	*Acp5, Adck3, Afap1l2, Apbb1, C2, Cd24, Cxcl12, Cyp4f6, Il27ra, Masp1, Ptpn6, RT1*-*DMb, Tf, Tgm2, Tinagl1, Tlr2, Vnn1, Zfr2*
				Down	*A2* *m, C3ar1, Ccl2, Ccl4, Ereg, F2rl1, Gch1, Il1rl1, Irf7, Irgm, Nppb, Oas1b, Oasl2, Prg4, Ptgs2, Rsad2*
	8	Cell surface receptor linked signal transduction (3) [25]	1.35	Up	*Adamts1, Adck3, Apoe, Boc, Cd24, Celsr1, Cxcl12, Disp1, Efna2, Efnb1, Fgfr2, Gli2, Gpr56, Lpar3, Ncam1, Plce1, Sfrp2, Tek, Tgfb1i1, Tgm2*
				Down	*Ctgf, Epha7, Fst, Itgb8, Ptger2*
N [12]	6	Nucleotide catabolic process (7) [12]	1.47	Up	*Akr7a3, Ampd3, Gucy1b3, Nt5e, Nudt7, Pde4a, Pde4b, Prodh*
				Down	*Gch1, Nppb, Ppat, Upp1*

Numbers in normal brackets denote the number of grouped GO categories. Absolute numbers of regulated genes per main group are given in squared brackets, examples of regulated genes are shown for up- and down-regulated genes

*H* hormone stimulus, *N* Nucleotide Catabolic Process

### Upstream regulation analysis identified more activation than de-activation

Upstream regulation analysis with IPA is based on gene expression patterns and predicts activation or deactivation of regulators of the differentially regulated genes. The results show that more upstream regulators are predicted to be activated (n = 51) than inhibited (n = 20).

These predictions are based on 220 genes from which 95 contributed to activation as well as to deactivation. The proportion of overall down- and up-regulation is mirrored in these genes with more down-regulation in inhibition as well as in activation (Tables 3, 4, 5, 6). The majority of deactivated upstream regulators (8 out of 20) are classified as transcription regulators. Activation is mainly predicted for cytokines (14 out of 51).

**Table 3:** Upstream regulator analysis with IPA: types of predicted upstream regulators

Activation (n = 51)		Inhibition (n = 20)	
Cytokines/group of cytokines	14	Transcription regulator	8
Others/complex of others	8	Cytokine	2
Kinases, group of kinases	8	Enzyme	2
Growth factors/complex of growth factors	6	Other	2
Transcription regulator	6	G-protein coupled receptor	1
Transmembrene receptors	4	Growth factor	1
Enzymes	3	Ligand-dependent nuclear receptor	1
Ligand-dependent nuclear receptor	1	Peptidase	1
Peptidase	1	Phosphatase	1
		Transporter	1

Summarizing the regulator according to their type revealed a high proportion of possibly activated cytokines, whereas transcription regulators play a major role in inhibition

Based on gene expression patterns, predictions are made on activation or inactivation of known upstream regulators. Absolute activation z-scores of higher than 2.0 are considered to be highly significant. We found more than twice as much regulators predicted to be activated as compared to inhibited. These tables show the predicted upstream regulators with an absolute z-score above 2.0 in detail—some are in fact complexes or groups. The prediction is opposed to the real measurement on the micro array (rightmost columns), as far as the respective genes have passed QC and is otherwise marked as “not measured”. Mean expression per group is given as logarithm of the intensity to base 2. Reasonably high expression values are in bold face. The column “regulation AR17” denotes if the respective gene is contained in the set of regulated genes (level = L1) or at least close to significance (level = L2/L3) which holds true for the minority of genes. Activation or inhibition is not necessarily reflected by significant change of gene expression, since processes not measurable on a micro array, like for example phosphorylation, are more likely to be responsible for that

**Table 4:** Upstream regulator analysis with IPA: proportion of up- and downregulated genes

Gene pattern	Activation only	Inhibition only	Both
Down regulation	64	28	50
Up regulation	28	5	45

The gene expression patterns upon which the prediction is made is constituted by both up-regulated and down-regulated genes. The predicted activation and inhibition is either based on two third down regulated (n = 114/n = 78) and one third upregulated genes (n = 73/n = 50). 50 downregulated genes and 45 upregulated genes contribute likewise to activation and inhibition (The details of the contributing gens are not shown here)

Based on gene expression patterns, predictions are made on activation or inactivation of known upstream regulators. Absolute activation z-scores of higher than 2.0 are considered to be highly significant. We found more than twice as much regulators predicted to be activated as compared to inhibited. These tables show the predicted upstream regulators with an absolute z-score above 2.0 in detail—some are in fact complexes or groups. The prediction is opposed to the real measurement on the micro array (rightmost columns), as far as the respective genes have passed QC and is otherwise marked as “not measured”. Mean expression per group is given as logarithm of the intensity to base 2. Reasonably high expression values are in bold face. The column “regulation AR17” denotes if the respective gene is contained in the set of regulated genes (level = L1) or at least close to significance (level = L2/L3) which holds true for the minority of genes. Activation or inhibition is not necessarily reflected by significant change of gene expression, since processes not measurable on a micro array, like for example phosphorylation, are more likely to be responsible for that

**Table 5:** Upstream regulator analysis with IPA: predicted activated regulators

IPA–prediction			Micro array analysis				
Upstream regulator	Molecule type	z-score	FDR	FC	Mean AR17	Mean noAR	Regulation AR17 [level]
*Ahr*	Ligand-dependent nuclear receptor	*3.185*	0.895	−1.017	−1.219	−1.194	
*Bmp6*	Growth factor	*2.791*	0.011	−1.483	*2.595*	*3.164*	
*Ccl5*	Cytokine	*2.190*	0.016	2.529	*2.922*	*1.583*	[Up L3]
*Ddx58*	Enzyme	*2.789*	0.019	2.096	*3.316*	*2.249*	[Up L3]
*Dock8*	Other	*2.530*	0.010	−1.656	*2.545*	*3.272*	[Down L2]
*Egf*	Growth factor	*2.539*		*<not measured>*			
*Erk:*	Group of kinases (n=7)	*2.372*			*<group>*		
*Mapk1*	Kinase		0.009	−1.019	*3.670*	*3.697*	
*Mapk3*	Kinase		0.027	−1.280	*5.646*	*6.002*	
*Mapk4*	Kinase		0.701	1.086	−2.321	−2.440	
*Mapk6*	Kinase		0.037	1.131	*5.152*	*4.975*	
*Mapk7*	Kinase		0.758	−1.047	*2.862*	*2.928*	
*Mapk12*	Kinase		0.018	−2.005	0.121	1.125	[Down L3]
*Mapk15*	Kinase		0.208	1.217	*0.507*	0.224	
*Mek:*	Group of kinases (n=7)	*2.942*			*<group>*		
*Map2k1*	Kinase		0.105	1.182	*4.651*	*4.410*	
*Map2k2*	Kinase		0.177	1.090	*4.816*	*4.691*	
*Map2k3*	Kinase		−1.066	−0.092	*4.360*	*4.452*	
*Map2k4*	Kinase		1.023	0.032	*3.336*	*3.303*	
*Map2k5*	Kinase		−1.058	−0.082	*3.327*	*3.408*	
*Map2k6*	Kinase		1.177	0.235	*3.760*	*3.525*	
*Map2k7*	Kinase		−1.125	−0.170	−1.445	−1.275	
*P38 Mapk:*	Group of kinases (n= 5)	*2.624*			*<group>*		
*Mapk1*	Kinase		0.009	−1.019	*3.670*	*3.697*	
*Mapk11*	Kinase		0.087	−1.343	0.139	*0.565*	
*Mapk12*	Kinase			*<see above>*			
*Mapk13*	Kinase			*<not measured>*			
*Mapk14*	Kinase		0.046	−1.276	*3.242*	*3.594*	
*Mapk2/1: group of*	Kinases (n= 2)	*2.401*			*<group>*		
*Map2k1*	Kinase			*<see above>*			
*Map2k2*	Kinase			*<see above>*			
*F7*	Peptidase	*2.592*		*<not measured>*			
*Fgf2*	Growth factor	*2.085*	0.122	1.178	0.417	0.180	
*Fos*	Transcription regulator	*2.086*	0.069	−1.425	*2.972*	*3.482*	
*Hras*	Enzyme	*3.258*		*<not measured>*			
*Ifn / Ifn alpha:*	Group of groups						
*Ifn:*	Group of cytokines	*2.429*			*<group>*		
*Ifn alpha:*	Group of cytokines	*2.228*			*<group>*		
*Ifna1*	Cytokine		0.104	1.306	*2.728*	*2.343*	
*Ifna2*	Cytokine	*2.448*		*<not measured>*			
*Ifna4*	Cytokine	*2.236*		*<not measured>*			
*Ifna5 - 8*	cyTokine (n=4)			*<not measured>*			
*Ifna10, 13, 14, 16, 17, 21*	Cytokine (n=6)			*<not measured>*			
*Ifnk*	Cytokine			*<not measured>*			
*Ifnw1*	Cytokine			*<not measured>*			
*Ifnz*	Cytokine			*<not measured>*			
*Ifn beta:*	Group of cytokines (n=2)	*2.767*					
*Ifnb1*	Cytokine	*2.591*	0.079	2.953	−1.949	−3.511	
*Il6*	Cytokine	*2.443*	0.014	1.481	−0.730	−1.296	
*Ifnar:*	Group of transmembrane receptors	*2.749*			*<group>*		
*Ifnar1*	Transmembrane receptor			*<not measured>*			
*Ifnar2*	Transmembrane receptor			*<not measured>*			
*Ifne*	Cytokine	*2.219*		*<not measured>*			
*Ifng*	Cytokine	*2.811*		*<not measured>*			
*Ifnl1*	Cytokine	*2.764*		*<not measured>*			
*Igf2*	Growth factor	*2.213*	0.001	−9.285	*1.909*	*5.124*	[Down L1]
*Ikbke*	Kinase	*2.090*	0.013	−2.034	−1.262	−0.238	[Down L3]
*Il1: group of*	Cytokines (n=11)	*2.207*					
*Il1b*	Cytokine			*<not measured>*			
*Il18*	Cytokine	*2.372*	0.300	−1.056	*0.531*	*0.610*	
*Il1f10*	Cytokine		0.689	1.102	−2.284	−2.424	
*Il1rn*	Cytokine		0.009	1.812	0.062	−0.796	[Up L2]
*Il33*	Cytokine		0.001	10.690	*1.998*	−1.420	[Up L1]
*Il17a*	Cytokine			*<not measured>*			
*Il36a*	Cytokine			*<not measured>*			
*Il36b*	Cytokine			*<not measured>*			
*Il36g*	Cytokine			*<not measured>*			
*Il36rn*	Cytokine		0.019	1.393	*2.076*	*1.598*	
*Il37*	Cytokine			*<not measured>*			
*Irf3*	Transcription regulator	*3.157*	0.520	1.336	*3.450*	*3.033*	
*Irf5*	Transcription regulator	*2.934*	0.113	1.096	*1.321*	*1.188*	
*Irf7*	Transcription regulator	*3.901*	0.003	5.540	*5.574*	*3.104*	[Up L1]
*Kras*	Enzyme	*2.616*	0.191	−1.097	*3.119*	*3.253*	
*Lh [Cga, Lhb]*	Complex	*2.012*					
*Lhb*	Other		0.480	1.044	*2.593*	*2.530*	
*Cga*	Other		0.251	1.273	−0.193	−0.541	
*Map3k7*	Kinase	*2.375*	0.352	−1.067	*4.577*	*4.671*	
*Mavs*	Other	*2.630*	0.021	−1.231	*2.569*	*2.868*	
*Pdgf bb*	Complex	*3.491*			<group>		
*Pdgfb*	Growth factor			1.156	*2.037*	*1.828*	
*Pdlim2*	Other	*2.324*	0.003	1.346	*3.226*	*2.798*	
*Samsn1*	Other	*2.309*		*<not measured>*			
*Sash1*	Other	*2.530*		*<not measured>*			
*Sphk1*	Kinase	*2.172*	0.611	1.237	−0.033	−0.341	
*Src*	Kinase	*2.158*	0.033	1.348	*3.704*	*3.273*	
*Stat1*	Transcription regulator	*2.194*	0.013	1.375	*3.163*	*2.703*	
*Stat2*	Transcription regulator	*2.173*	0.535	1.067	*3.865*	*3.772*	
*Tac1*	Other	*2.153*	0.910	1.055	−2.385	−2.462	
*Tgfa*	Growth factor	*2.165*	0.586	1.088	*2.152*	*2.031*	
*Ticam1*	Other	*2.702*	0.574	−1.035	*3.646*	*3.696*	
*Tlr3*	Transmembrane receptor	*3.633*	0.049	−1.414	−0.244	0.256	
*Tlr4*	Transmembrane receptor	*3.175*		*<not measured>*			
*Tlr9*	Transmembrane receptor	*3.645*	0.249	1.134	*2.534*	*2.353*	
*Tnfsf11*	Cytokine	*2.168*	0.539	1.115	−0.643	−0.800	

Z-score < 2.0

Based on gene expression patterns, predictions are made on activation or inactivation of known upstream regulators. Absolute activation z-scores of higher than 2.0 are considered to be highly significant. We found more than twice as much regulators predicted to be activated as compared to inhibited. These tables show the predicted upstream regulators with an absolute z-score above 2.0 in detail—some are in fact complexes or groups. The prediction is opposed to the real measurement on the micro array (rightmost columns), as far as the respective genes have passed QC and is otherwise marked as “not measured”. Mean expression per group is given as logarithm of the intensity to base 2. Reasonably high expression values are in bold face. The column “regulation AR17” denotes if the respective gene is contained in the set of regulated genes (level = L1) or at least close to significance (level = L2/L3) which holds true for the minority of genes. Activation or inhibition is not necessarily reflected by significant change of gene expression, since processes not measurable on a micro array, like for example phosphorylation, are more likely to be responsible for that

**Table 6:** Upstream regulator analysis with IPA: Predicted inactivated regulators

IPA–prediction			Micro array analysis				
Upstream regulator	Molecule type	z-score	FDR	FC	Mean AR17	Mean noAR	Regulation AR17 [level]
*Ackr2*	G-protein coupled receptor	*−3.162*	0.061	1.308	0.389	0.001	
*Bcl6*	Transcription regulator	*−2.353*	0.233	1.041	*1.031*	*0.973*	
*Fbxo32*	Enzyme	*−2.213*	0.797	1.048	−0.588	−0.655	
*Gata2*	Transcription regulator	*−2.965*	0.061	−3.356	−1.682	0.065	
*Gdf2*	Growth factor	*−2.400*		*<not measured>*			
*Hmox1*	Enzyme	*−2.425*	0.011	1.631	*3.108*	*2.402*	[Up L3]
*Htt*	Transcription regulator	*−2.828*	0.560	1.033	*2.380*	*2.334*	
*Il10*	Cytokine	*−2.394*		*<not measured>*			
*Il1rn*	Cytokine	*−3.108*	0.009	1.812	0.062	−0.796	[Up L2]
*Irgm1*	Other	*−2.236*		*<not measured>*			
*Mitf*	Transcription regulator	*−2.535*	0.081	−1.456	*2.487*	*3.029*	
*Nkx2-3*	Transcription regulator	*−2.183*	0.168	1.119	−1.622	−1.785	
*Pparg*	Ligand-dependent nuclear receptor	*−2.353*	0.009	−1.950	−0.203	0.761	[Down L2]
*Runx2*	Transcription regulator	*−2.137*	0.021	1.358	*4.291*	*3.850*	
*Sftpa1*	Transporter	*−2.111*	0.752	−1.087	−2.019	−1.899	
*Shh*	Peptidase	*−2.168*		*<not measured>*			
*Socs1*	Other	*−3.084*		*<not measured>*			
*Socs3*	Phosphatase	*−2.216*	0.591	1.111	−1.131	−1.283	
*Sox9*	Transcription regulator	*−2.219*		*<not measured>*			
*Trim24*	Transcription regulator	*−2.331*	0.119	−1.166	*2.191*	*2.413*	

Z-score < −2.0

Based on gene expression patterns, predictions are made on activation or inactivation of known upstream regulators. Absolute activation z-scores of higher than 2.0 are considered to be highly significant. We found more than twice as much regulators predicted to be activated as compared to inhibited. These tables show the predicted upstream regulators with an absolute z-score above 2.0 in detail—some are in fact complexes or groups. The prediction is opposed to the real measurement on the micro array (rightmost columns), as far as the respective genes have passed QC and is otherwise marked as “not measured”. Mean expression per group is given as logarithm of the intensity to base 2. Reasonably high expression values are in bold face. The column “regulation AR17” denotes if the respective gene is contained in the set of regulated genes (level = L1) or at least close to significance (level = L2/L3) which holds true for the minority of genes. Activation or inhibition is not necessarily reflected by significant change of gene expression, since processes not measurable on a micro array, like for example phosphorylation, are more likely to be responsible for that

### Validation of microarray data by RT-qPCR

**Fig. 4:**
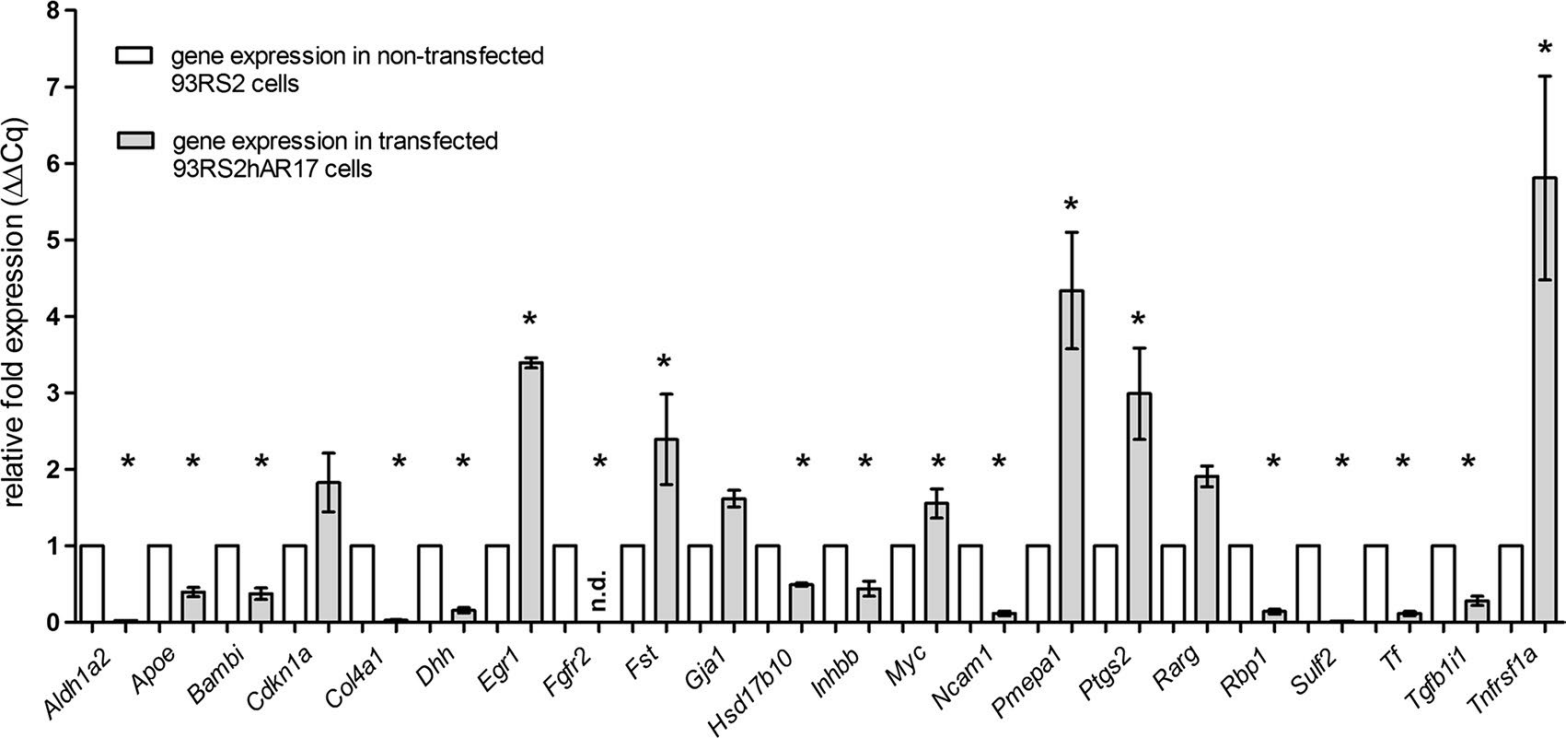
Quantitative RT-PCR was performed to validate microarray analysis results. Gene expression analysis for 22 genes that showed deviant gene expression in microarray analysis has been performed using 2^−ΔΔCq^ method. RT-qPCR has been performed using three technical replicates in a double determination. Gene expression in non-transfected 93RS2 cells was used as calibrator and therefore set as “1”. Data are presented as mean ± SEM. (standard error of the mean) and differences in mean values have been assessed with SPSS software; *p ≤ 0.05, *n.d.* not detectable

For validation of microarray results we performed RT-qPCR for 22 candidate genes, showing different ranges of regulation (up, down). Among the chosen genes, some are mainly associated with development and are known Sertoli cell markers, such as *Dhh* [20], *Gja1* [21], *Inhbb* [22], and *Tf* [23]. Other genes are markers for differentiation and proliferation (e.g. *Bambi* and *Tgfb1i1* [24]) and some are involved in apoptosis, such as *Myc* and *Tnfrsf1a* [25]. Results from RT-qPCR were mostly consistent with data from microarray analysis (Fig. 4). Relative gene expression was lower in transfected compared to non-transfected Sertoli cells in 13 of 22 cases. Gene expression of *Cdkn1a*, *Egr1*, *Fst*, *Gja1*, *Myc*, *Pmepa1*, *Ptsg2*, *Rarg* and *Tnfrsf1a* was higher in 93RShAR17 cells compared to *Ar*-deficient 93RS2 cells. In the latter case, it has to be mentioned, that differences of the means did not reach significance in four genes, due to high variability of C_q_.

## Discussion

To study the effects of androgens and AR/*Ar* on diverse cell culture systems and the relevance for cell biology, cell culture experiments were conducted in different human cell lines (e.g. breast cancer cells, adrenocortical carcinoma cells, murine skeletal muscle cells or liver carcinoma cells [9–12]). Also AR-deficient cell lines have been used, either transfected with AR [14, 15] or without [13]. Both groups working with transfected cell lines performed their experiments using either not stimulated [14] or mock-transfected cells [15] as negative controls. Moreover, Jacobsen et al. [26] showed, that transfection of MCF-7 breast cancer cells lead to severe differences in gene expression levels in distinct genes, depending on the transfection reagent used. Interestingly, transfection with a vector encoding for a reporter gene and a vector without insert, respectively, revealed no differences in gene expression. This implies, that the transfection procedure itself might alter gene expression in these cells. Therefore, we performed gene expression analysis with AR-transfected rat Sertoli cells using non-transfected cells as controls to show “intrinsic” gene expression alterations due to the transfection procedure. As electroporation has been shown to be superior with respect to cell viability and also transfection efficiency compared to chemical transfection using lipofectamine [27], we applied this technique to introduce the AR. Cell viability was not influenced by electroporation, but whole genome microarray analysis showed an altered gene expression. Surprisingly, more genes have been down-regulated than up-regulated comparing transfected and non-transfected cells. We selected 22 genes showing an altered expression pattern and confirmed microarray results with RT-qPCR analysis. In the following, we will discuss in more depth interesting genes and pathways, respectively.

Among the down-regulated genes, many are involved in metabolic processes, as for example in iron transport and metabolism (cytochrome b reductase 1 (*Cybrd1)*, FC = −107; transferrin (*Tf*), FC = −6,898; six-transmembrane epithelial antigen of the prostate 2 (*Steap2*), FC = −2.3). Iron is relevant for Sertoli cells in two different aspects: as supervisors of germ cell development, Sertoli cells provide iron which is needed for DNA synthesis and cell growth of germ cells that undergo multiple mitotic divisions [28]. On the other hand, Sertoli cells avoid toxic environmental conditions that might be given at elevated concentrations of insoluble ferric iron (Fe^3+^). Therefore, Sertoli cells secrete transferrin, the product of the *Tf* gene [29], which may be used as a marker for Sertoli cell function and differentiation [23] as it creates an environment low in free iron that impedes bacterial survival in a process called iron withholding. The protein level of *Tf* decreases in inflammation. The lower expression of *Tf* gene, which was confirmed in RT-qPCR (Fig. 4), could be interpreted as a sign of severe disturbance and inflammation of cells.

The latter is reflected by the high proportion of upstream regulators related to immune response (= cytokines and members of the MAP kinase signalling pathway) that are predicted to be activated (Table 3) and the presence of multiple immune response-related genes on top of the list in up-regulation (Table 1). “Immune response” is the second huge cluster of altered genes in our study, represented by e.g. prostaglandin-endoperoxidase synthase 2 (*Ptgs2*, FC = 3.558) also known as cyclooxygenase 2 (*Cox2*). An increase in *Cox2* expression was observed by Matzkin et al. [30] in Leydig cells of infertile men showing either hypospermatogenesis, Sertoli cell only syndrome or maturational arrest. By increased numbers of testicular macrophages, levels of interleukin 1β (*Il*-*1β*) are increased and activates *Ptgs2*, the key enzyme in prostaglandin synthesis culminating in inflammation. The expression of *Tf*, *Ptgs2* and interleukins is coupled in Sertoli cells; as shown by Yamaguchi et al. [31], incubation with cisplatin lead to an increase in *Ptgs2* and a decrease in *Tf* expression in Sertoli cell cultures, similar to our study. Additionally, an analysis of upstream regulation using IPA revealed a high number of key players in inflammation to be activated showing congruently high FCs for *Ccl5*, *Irf7*, and *Ifnb1*. This might on the one side be due to the transfection procedure itself and/or reflect inflammatory processes in the cells due to increased cell damage. Remarkably, an influence of molecular biological techniques on gene expression and immune response has been observed also in regard to short-interfering RNAs (siRNAs). Sledz et al. reported an induction of interferon β levels in a human glioblastoma cell line which was transfected with siRNAs as a non-specific side effect additionally to the silencing of the target gene lamin [32] .

**Fig. 5:**
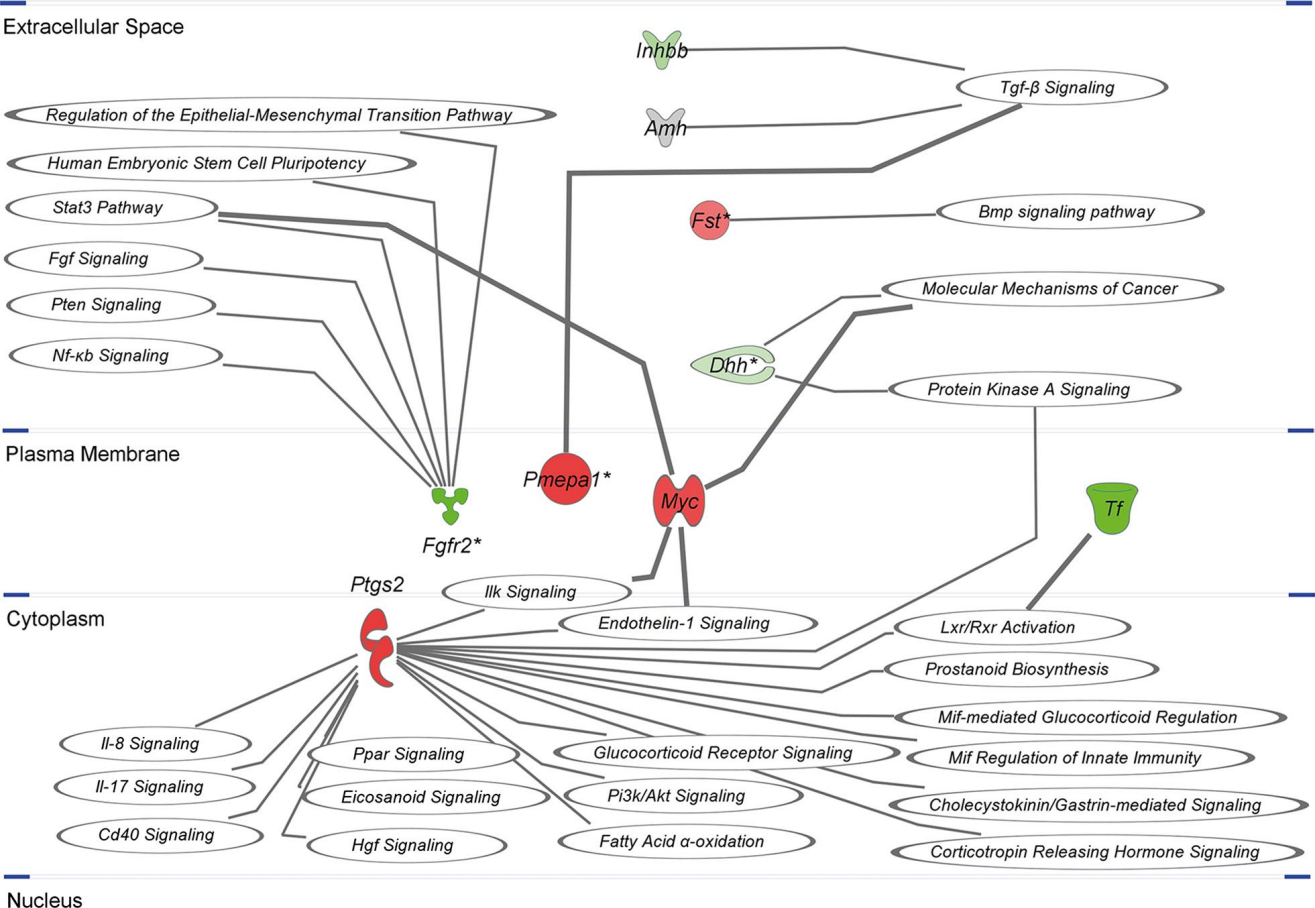
Illustration of eight genes and their association to known pathways in IPA. *Green color* denotes down-regulation, whereas *red color* denotes up-regulation

Not only metabolism and immune response gene expression seem to be altered in transfected Sertoli cells, but also cell cycle and development genes (desert hedge hog (*Dhh*) FC = −2.032; fibroblast growth factor receptor 2 (*Fgfr2)* FC = −8.239; follistatin (*Fst*) FC = 2.162; inhibin beta b (*Inhbb*) FC = −3.126). *Dhh* is involved in various areas of embryonic development, including testicular cord formation. It is expressed in mouse Sertoli cell precursors during mid- to late gestation [33] and also is important for germ cell development after puberty in mouse [34] and rat testis [35]. In the mouse, a lack of *Dhh* results in a severe impairment of spermatogenesis due to a lack of spermatogonial development beyond primary spermatocytes [34]. *Fgfr2* is a known differentiation factor in prenatal Sertoli cells as it is concomitantly expressed with *Sry* and is essential for subsequent expression of anti-muellerian hormone (*Amh*) and *Sox9* [36]. IPA analysis of upstream regulation predicted an inhibition of transcription factor *Sox9* with a z-score of −2.2 (Table 6). Moreover, lack of *Fgfr2* might cause a partial XY sex reversal, as loss of *Fgfr2* leads to an up-regulation of Follistatin (*Fst*), a female somatic cell marker [37], which was confirmed by microarray and RT-qPCR. A down-regulation of the Sertoli cell marker *Inhbb* (for review see [38]) also points to a decreased Sertoli cell function and a severe disturbance of spermatogenesis in the rat [39]. Figure 5 shows the association of *Inhbb*, *Fst*, *Dhh*, *Pmepa1*, *Fgfr2*, *Ptgs2*, *Tf* and *Myc* as especially interesting genes on known pathways as predicted by IPA.

**Fig. 6:**
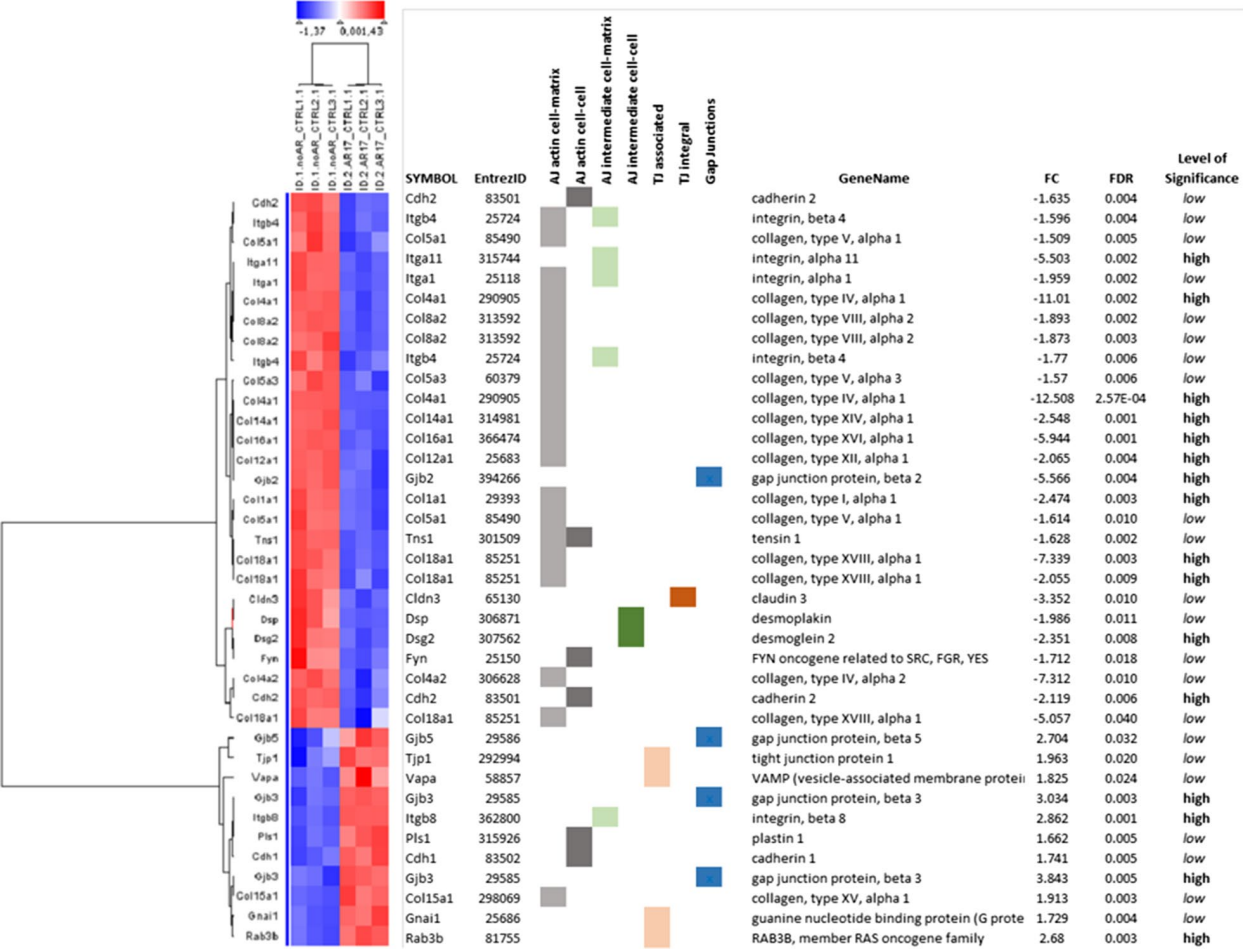
Hierarchical clustering of significantly regulated genes involved in cell adhesion. Clustering was done using “Pearson correlation” and “complete linkage”. The tree on the left reflects the distances between gene profiles based on this algorithm. AJ actin/intermediate = adherents junctions based on actin or intermediate filaments, TJ = tight junctions. Low significance: 1.5 < FC < 2.0 and/or FDR 0.01–0.05 High significance: FC > 2.0 and FDR < 0.01

A disturbance of Sertoli cell function is also visible in gene expression alteration concerning the functional cluster “Cell adherence” or “Cell adhesion” (Fig. 6, e.g. collagen type IV alpha (*Col4a1*) FC = −12.503; gap junction protein 1 (*Gja1)* FC = −1.188). Cell adhesion and formation of tight junctions between Sertoli cells generating the blood-testis barrier is one of the most important features of Sertoli cell maturation and function (for review see [22]) as it is a prerequisite for intact spermatogenesis. Also cell-to-cell contact and communication seem to be disturbed in transfected cells as indicated by the down-regulation of *Gja1*, also known as connexin 43 (for review see [20]).

## Conclusion

Our results indicate a severe disturbance of Sertoli cell metabolism, function and cell biology concerning immune status and generation of blood-testis barrier, caused by the transfection procedure even without androgen stimulation of cells. The alterations in gene expression levels might either be related to the transfection procedure itself and/or to the insertion of human AR into *Ar*-free rat Sertoli cells. A microarray analysis with mock-transfected Sertoli cell line would be needed to distinguish both possibilities. We consider the altered gene expression to be caused by AR insertion, as many of the altered genes were identified as AR and Sertoli cell specific. In either case, incubation of transfected cell lines with testosterone or dihydrotestosterone might lead to false-positive or false-negative results and additionally, also non-genomic pathways including AR/*Ar* action may be altered by transfection procedures. Therefore, suitable negative controls are needed for stimulation experiments with T or DHT, i.e. non-transfected cells as appropriate negative controls. Gene expression has to be normalized by these non-transfected cells to avoid false-positive or false-negative results regarding gene regulation.

## Methods

### Cell lines and culture conditions, human positive control tissue

We determined the expression of *Ar* in different Sertoli cell lines by RT-PCR. For this study, we used four existing immortalized Sertoli cell lines from either mouse (WL3, SK-11) [40, 41] or rat testis (93RS2) [18] kindly provided by our collaborators. Additionally, SCIT-C8 cells were generated from immortalized Sertoli cells from rat testis as described by Konrad et al. [42]. We did not conduct any animal research in our study and therefore ethics approval was not required. Total RNA of these cells was isolated by peqGold Total RNA Kit (Peqlab, Erlangen, Germany), set to a concentration of 200 ng/µl and genomic DNA was digested by RNase-Free DNase Set (Qiagen, Hilden, Germany). Reverse transcription was performed with Omniscript RT Kit (Qiagen). The mastermix was prepared as follows: 2 µl Buffer RT (10×), 2 µl dNTP mix (5 mM each), 0,7 µl RNAse inhibitor (20 units/µl, Invitrogen via LifeTechnologies, Carlsbad, CA, USA), 0,2 µl Oligo-dT primer (10 µM, Qiagen) and 1 µl Omniscript Reverse Transcriptase were mixed and RNase free water was added to a final volume of 10 µl. To test genomic DNA digestion success, we omitted reverse transcriptase and replaced it by RNase free water for one reaction. After addition of 1 µl RNA (200 µg/µl), we incubated the reaction mix for 1 h at 37 °C. cDNA not directly used for further experiments was stored at −20 °C. Amplification of *Ar* was achieved with a matching primer pair for murine and rat *Ar* obtained from Eurofins MWG Operon (Huntsville, AL, USA) as can be seen in Table 7 and *Taq* PCR Master Mix Kit (Qiagen). Mastermix was prepared as follows: 34 µl *Taq* PCR Master Mix, forward and reverse primer (2,5 µl each) and RNase free water as well as 1 µl cDNA were mixed to a final volume of 50 µl. Amplification was performed with 1× 94 °C for 4 min, 35× (94 °C for 40 s, 60 °C for 45 s, 72 °C for 90 s) and 1× 72 °C for 5 min.

As the prepubertal rat Sertoli cell line 93RS2 proved to be devoid of *Ar*, we chose this cell line for further experiments. The cells were maintained in a 5 % CO_2_ atmosphere at 34 °C. The standard culture media consists of DMEM high glucose mixed 1:1 with Ham’s F-12 media plus 100 units/ml penicillin, 0,1 mg/ml streptomycin, 10 % FBS-Gold (total protein 3.0–4.5 g/dl), and 1 % ITS (1000 mg/l Insulin, 550 mg/l Transferrin, 0.68 mg/l Selenin). Unless otherwise stated, cell culture media were purchased from Invitrogen (via Life Technologies, Carlsbad, CA, USA).

### Ethics, consent and permissions

For positive control used in RT-PCR and Western Blotting, we used testis homogenate from a patient showing normal spermatogenesis attending the andological clinic in Münster for re-fertilization surgery. After written informed consent, biopsies were taken under general anesthesia. The reported study has been approved by the Ethics committee of the Medical Faculty of the Justus Liebig University Giessen (decision 75/00 and 56/05).

### AR transfection in 93RS2 Sertoli cells

We introduced a commercial available full length human AR (OriGene, Rockville, MD, USA), containing 17 CAG triplets, into the expression vector pcDNA 6.2 C-EmGFP (Invitrogen) after amplification of AR using GC-Rich PCR System (Roche, Basel, Switzerland), according to manufacturer’s instructions. Transfection of 93RS2 cells was performed using the microporation system MP-100 (Peqlab). Cells were detached by Trypsin (PAA, Piscataway, NY, USA) and subsequently adjusted to 200,000 cells per well in a 6-well-plate. After re-suspending the cells in the provided buffer, plasmid DNA was added. We used a current strength of 1150 V for 20 ms with two pulses.

### Validation of transfection success in 93RS2 by immunofluorescence, RT-PCR and Western Blotting

24 h after transfection, transfected cells (93RShAR17) were fixed in 6-well-plates with 4 % paraformaldehyde for 20 min at room temperature, washed three times with PBS and permeabilized with 0.1 % Triton x-100. After transferring the cells to a 12-well-plate and washing with PBS, unspecific binding sites were blocked with 3 % BSA (bovine serum albumin, Carl Roth GmbH + Co.KG, Karlsruhe, Germany) in TBST (Tris-Buffered Saline and Tween 20, Carl Roth) and washed again with PBS. The rabbit anti-GFP antibody (ab290, Abcam, Cambrigde, UK) was added in a dilution of 1:200. After incubation for 3 h and washing with PBS, goat anti-rabbit Alexa 488 antibody (Invitrogen) was added in a dilution of 1:200. After a final incubation for 1 h in the dark, cells were washed and embedded with Vectashield mounting medium with DAPI (H-1200, Vector Laboratories, Dossenheim, Germany). Transfection efficiency was evaluated using a fluorescence microscope (AxioPhot, Zeiss, Oberkochen, Germany). Western Blot analysis to prove antibody specificity and AR protein expression in transfected Sertoli cells was performed as described elsewhere [43]. Shortly, proteins extracted from cell lysates of transfected 93RS2 cells and human testis tissue were submitted to protein extraction using TRI Reagent^®^ RNA Isolation Reagent (Sigma-Aldrich, St. Louis, MO, USA) according to Chomczynski [44]. Proteins were run on a 3–8 % Tris–acetate gel (Life Technologies, Carlsbad, CA, USA) for 75 min at 150 V and blotted on nitrocellulose membrane for 75 min at 30 V. A polyclonal rabbit anti-human AR antibody (sc-816, Santa Cruz Biotechnology Inc., Dallas, TX, USA) in a 1:500 dilution and a biotinylated goat anti-rabbit antibody (E0432, Dako, Glostrup, Denmark) in a 1:1000 dilution were used. As weight marker, we used HiMark™ Pre-Stained Protein Standard (Life Technologies). Signal detection was performed by incubating the membrane with Vectastain Elite ABC Standard Kit (Vector Laboratories, Inc., Burlingame, CA, USA) and TrueBlue™ Peroxidase Substrate (KPL, Gaithersburg, MD, USA). A negative control was performed by omitting the primary antibody.

To detect AR mRNA in transfected cells, we performed RT-PCR (primers may be seen in Table 7) as described earlier with minor changes concerning the cycling conditions: 1× 94 °C for 4 min, 35× (94 °C for 45 s, 55 °C for 45 s, 72 °C for 90 s) and 72 °C for 5 min resulting in a 591 bp amplicon. The CAG repeat length was confirmed using RT-PCR with subsequent high resolution polyacrylamide gel electrophoresis (PAGE) as described recently [45].

**Table 7:** Primer sequences

Primer name	GenBank accession no.	Sequence (5′ ≥ 3′)		Amplicon length (bp)	RT-qPCR efficiency (%)
*Ar*	NM_013476	For	CACATCCTGCTCAAGGCGCTT	181	n.a.
	(mouse)	Rev	CCCAGAAAGGATCTTGGGCAC		
	NM_012502			181	n.a.
	(rat)				
AR	NM_000044	For	TATCCCAGTCCCACTTGTG	592	n.a.
		Rev	TCTCTCCCAGTTCATTGAGG		
*Aldh1a2*	NM_053896	For	TCAGACTTCGGGCTTGTAGC	125	94.3
		Rev	GGGCTCTGAGCATTTAAGGC		
*Apoe*	NM_001270681	For	TGATGGAGGACACTATGACG	188	105.8
		Rev	CATGGTGTTTACCTCGTTGC		
*Bambi*	NM_139082	For	CCATGCCCACTTTGGAATGC	126	128.0
		Rev	TTCTGCTGCTGTCATGCTGG		
*Cdkn1a*	NM_080782	For	CACAGGAGCAAAGTATGCCG	125	135.1
		Rev	GCGAAGTCAAAGTTCCACCG		
*Col4a1*	NM_0011350009	For	GGAGAACCTGGCAGTGATG	118	99.9
		Rev	CACCCTTGGAACCTTTGTC		
*Dhh*	NM_053367	For	TTGGCACTCCTGGCACTATC	124	102.2
		Rev	CGGGCATACTAGGCACAAAC		
*Egr1*	NM_012551	For	GTGGGAGAAAGTTTGCCAGG	125	111.3
		Rev	GTAGGAAGAGAGGGAAGAGG		
*Fgfr2*	NM_012712	For	CAGCTTCCCCAGATTACCTG	92	94.4
		Rev	CATTCGGCAAAAGATGACTG		
*Fst*	NM_012561	For	TCCAGTACCAGGGCAAATG	78	96.2
		Rev	TCTGATCCACCACACAAGTG		
*Gja1*	NM_012567	For	GTACGGGATTGAAGAGCACG	119	105.5
		Rev	TGTACCACTGGATGAGCAGG		
*Hsd17b10*	NM_031682	For	GAGGAAACTGCATATTTGCC	106	110.5
		Rev	TTGACAGCCACATCTATACG		
*Inhbb*	NM_080771	Rev	ACGGGTCAAGGTGTACTTCC	96	100.3
		For	AAGGTATGCCAGCCACTACG		
*Myc*	NM_0123603	Rev	TACATCCTGTCCGTTCAAGC	67	108.0
		For	GCCGTTTCCTCAGTAAGTCC		
*Ncam1*	NM_031521	Rev	ACGATGATGACTCCTCTACC	150	94.1
		For	GCGCATTCTTGAACATGAGC		
*Pmepa1*	NM_001107807	Rev	TGGTGATGGTGGTGATGATC	76	134.2
		For	CTGTGTCGGCTGATGAAGG		
*Ptsg2*	NM_017232	Rev	ACCGTGGTGAATGTATGAGC	104	98.4
		For	TCTTGTCAGAAACTCAGGCG		
*Rarg*	NM_001135249	Rev	TCACCAAGGTCAGCAAAGCC	125	141.9
		For	ACTGAACTTGTCCCACAGCC		
*Rbp1*	NM_012733	Rev	CTTCAGTGTGTTCAGAAGGG	117	87.9
		For	CTTGAACACTTGCTTGCAGG		
*Rplp2*	NM_001030021	Rev	TTGCCTCTTATCTGCTGGCC	110	103.4
		For	GTTGAGTCGTTCATCGTCCG		
*Sulf2*	NM_001034927	Rev	TTCCTGCCCAAGTATCAGC	108	111.5
		For	CCCAGAAGCGTCCTCTACAC		
*Tf*	NM_001013110	Rev	TGAGGTCTTGCCACAGAAGG	125	102.4
		For	CCACAACAGCATGAGAAGGG		
*Tgfb1i1*	NM_001191840	Rev	ACTACATCTCGGCACTCAGC	101	106.5
		For	ACCCTCGTGCTCAAAGAAGC		
*Tnfrsf1a*	NM_013091	Rev	AAAGAGGTGGAGGGTGAAGG	128	101.7
		For	ACAGGATGACTGAAGCGTGG		
*Ubc*	NM_017314	Rev	GGCAAAGATCCAGGACAAGG	100	99.4
		For	TTGTAGTCTGACAGGGTGCG		

Sequence and RT-qPCR efficiency of primers used for the study

*n.a.* not applied

### RNA isolation for microarray analysis

Total RNA of transfected 93RShAR17 cells as well as of non-transfected 93RS2 cells (using three technical replicates (N1-N3) each) was extracted using the peqGold total RNA kit (Peqlab) following manufacturer’s instructions. The amount of RNA was measured on a BioPhotometer (Eppendorf, Hamburg, Germany) as follows: 93RS2 N1 2200 ng/µl, N2 2130 ng/µl and N3 1920 ng/µl and 93RS2hAR17 N1 990 ng/µl, N2 1150 ng/µl and N3 1065 ng/µl (each replicate with a total volume of 15 µl). RNA was stored after extraction until use at −80 °C and transported in liquid nitrogen. The quality of total RNA was checked on a 1 % agarose gel stained with ethidium bromide (Sigma-Aldrich) as well as on Agilent 2100 Bioanalyzer using Eukaryote Total RNA Nano Assay (Agilent Technologies, Santa Clara, CA, USA). For this purpose, RNA was diluted to a concentration of 300 ng/µl. Only high quality RNA samples were used for microarray analysis.

### Microarray analysis

#### cRNA synthesis and hybridization

Extracted RNA was transcribed into biotinylated cRNA using MessageAmp™ II-Biotin *Enhanced* Kit (LifeTechnologies). Biotinylated cRNA again was quality checked on Agilent 2100 Bioanalyzer as stated above followed by cRNA fragmentation and finally hybridization on CodeLink Rat Whole Genome using the CodeLink Expression Assay Kit (GE Healthcare, Chalfont St. Giles, Buckinghamshire, UK). For this, 10 µg cRNA was diluted with nuclease-free water to final volume of 20 µl and mixed with 5 µl fragmentation buffer (taken from CodeLink iExpress iAmplify cRNA Prep & Hyb Kit, GE Healthcare) and fragmented at 94 °C for 20 min and subsequent cooling to 0 °C on ice. Hybridization solution was prepared by mixing hybridization buffer component A and B (taken from CodeLink iExpress iAmplify cRNA Prep & Hyb Kit), nuclease-free water and 25 µl fragmented cRNA. Denaturation of cRNA was performed at 90 °C for 5 min with subsequent cooling on ice. Hybridization reaction was carried out at 37 °C for 18 h. Subsequent washing was performed with 0.75 × TNT (1 M Tris–HCl, 5 M NaCl and 20 % Tween 20) buffer. Bioarrays were stained with Cy5™-streptavadin (GE Healthcare) and scanned using the GenePix^®^ 4000 B scanner and the GenePix Pro 4.0 Software (Axon Instruments, Arlington, USA). Scan resolution was set to 5 microns. A total of 2 × 3 = 6 array images were subjected to data analysis. Spot signals of CodeLink bioarrays were quantified using the CodeLink System Software 5.0.0.31312 which generated local background corrected raw as well as median centred intra-slide normalized data.

#### Quality control of microarray data

The genes represented by probe sets were annotated using the biocLite package (BioConductor) with the library “rwgcod.db” for CodeLink Rat Whole Genome arrays. The intra-slide normalized data containing 35129 rows and 6 columns (200 k values) were processed by an automated workflow that includes omission of control genes (n = 1280), removal of genes with poor QC (n = 1300 values, 0.6 %) or negative sign (n = 1603 values, 0.8 %), removal of probe sets with too high proportion (≥50 %) of missing values per group (n = 203 probe sets, 0.5 %) or with not any group having at least 50 % of values flagged as “G = good” and 50 % values above threshold (n = 7177 probe sets, 21.2 %), removal of outliers (expression values deviating more than fourfold from the group median, n = 427 values, 0.3 %). A total of 26452 probe sets remained after quality control with 1257 probe sets (=4.7 %) containing 1235 missing values (=0.8 %).

Remaining missing values were imputed by probabilistic principal component analysis (PPCA) using the R-package pca Methods. Imputed dataset was quantile normalized using the R-package limma [46], and logarithm for the base 2 was calculated.

#### Differential gene expression

Students *t* test was applied and a false discovery rate (FDR) ≤0.01 was set for the significance level with an absolute fold change (FC) ≥2 between transfected and non-transfected cells.

#### Functional gene analysis: overrepresentation analysis

Enriched functional gene ontology (GO) categories within the differentially regulated genes were determined using DAVID version 6.7 [47, 48]. Functional annotation clustering as well as an enrichment score was calculated for each cluster.

#### Upstream regulation analysis

To identify the regulators responsible for the observed gene expression profiles, we performed prediction analysis for activation or inhibition of upstream regulators using the Ingenuity^®^ Pathway Analyzer and the Ingenuity^®^Knowledge Base (IPA, Qiagen). Prediction is given as a z-score with >2 for activated and <2 for inactivated upstream regulators.

#### Validation of microarray results by quantitative RT-PCR (RT-qPCR)

For validation of microarray data, we performed RT-qPCR with 93RShAR17 and non-transfected 93RS2 cells for 22 genes (Table 7) that have been shown to be significantly altered in microarray analysis. All primer pairs obtained from MWG Operon have been validated in standard RT-PCR using rat testis as positive control. For this pupose, total RNA from rat testis was extracted using TRI Reagent^®^ RNA Isolation Reagent (Sigma-Aldrich) according to Chomczynski [44]. Genomic DNA was digested by using DNase I (Roche). For this, 6,65 µl RNA (200 ng/µl) were incubated with 1 µl MgCl_2_ (25 mM, Thermo Fisher Scientific), 1 µl DNase Buffer (Roche), 0,25 µl RNase inhibitor (40 units/µl, Thermo Fisher Scientific) and 1 µl DNase I for 25 min at 37 °C in a thermocycler. After a enzyme heat inactivation for 5 min at 75 °C, RNA was immediately reversely transcribed into cDNA. For this, 1,5 µl DNase-treated RNA was mixed with 1 µl 10x PCR Gold Buffer, 2 µl MgCl_2_ (25 mM), 1 µl dNTP mix (each 2,5 mM), 0,5 µl random hexamer primer (50 mM), 0,5 µl RNase inhibitor (20 units/µl), 0,5 µl MultiScribe^®^ Reverse Transcriptase (50 units/µl) and RNase free water to a final volume of 9 µl. All reagents were obtained from Thermo Fisher Scientific. For -RT control, reverse transcriptase was replaced by the same amount of RNase free water. Incubation was performed as follows: 8 min at 21 °C, 15 min at 42 °C and 5 min at 99 °C. cDNA was stored at −20 °C until use. For primer validation in standard RT-PCR, 1 µl cDNA was mixed with 2,5 µl 10× PCR Gold Buffer, 2 µl MgCl_2_ (25 mM), dNTP mix (each 2.5 mM), 1 µl forward and reverse primer, respectively (each 10 pM), 0.125 µl AmpliTaq Gold^®^ DNA Polymerase (5 units/µl) and RNase free water to a final volume of 25 µl. Cycling conditions were: 1× 94 °C for 9 min, 35× (94 °C for 45 s, 60 °C for 45 s, 72 °C for 45 s) and 72 °C for 5 min. Length of the resulting amplicons was checked in an agarose gel electrophoresis as described earlier. For RT-qPCR dilution series we used rat *Rplp* and *Ubc* as internal reference genes and performed triple determination in a decreasing 10- fold dilution series (undil., 1:10, 1:100). RT-qPCR efficiency (E) has been calculated using Bio-Rad CFX Manager version 3.1 (Bio-Rad) from the standard curve’s slope and may be seen in Table 7. Reference genes have been determined by using a TaqMan^®^ Array Rat Endogenous Control Plate (96-well, 32 reference genes pre-plated, Applied Biosystems via Thermo Fisher Scientific, Waltham, MA, USA).

For RT-qPCR, total RNA from transfected and non-transfected cells was extracted using peqGold Total RNA Kit (PEQlab) and reversely transcribed into cDNA as described above. As technical replicates we used cell pellets from three independent passages and for each specimen, double determination was performed using 1 µl of cDNA, 4 µl EvaGreen mastermix (no Rox) (Bio&Sell, Feucht, Germany), 0.6 µl forward and reverse primer each and 12.8 µl sterile aqua bidest to a final volume of 20 µl. RT-qPCR conditions were 1× 95 °C for 15 min, 40× (95 °C for 15 s, 60 °C for 30 s, 72 °C for 20 s) followed by melt curve analysis (1× 95 °C for 10 s, 65 °C to 95 °C, increment 0.5 °C for 5 s) on a CFX96 RealTime cycler (Bio-Rad Laboratories, Hercules, CA, USA). Relative gene expression was calculated by the 2^−ΔΔCq^ method, using *Rplp* and *Ubc* as internal reference genes. Expression levels represent x fold higher expression in the transfected than in the non-transfected cells (set as “1”). For statistical analysis, differences of the mean were assessed by ANOVA analysis. P-values of p ≤ 0.05 are set as statistically significant. The C_q_ values for all transcripts may be seen in Additional file 1: Table S1.

## Availability of supporting data

Complete microarray data may be found on GEO Accession Viewer database [19] with accession number GSE57653. Single Sertoli cell line data may be found under accession numbers GSM1385418 (Sertoli Cell Line noAR_1), GSM1385419 (Sertoli Cell Line noAR_2), GSM1386001 (Sertoli Cell Line noAR_3), GSM1385420 (Sertoli Cell Line AR17_1), GSM1385421 (Sertoli Cell Line AR17_2), GSM1385422 (Sertoli Cell Line AR17_3). Raw data of RT-qPCR experiments can be seen in Additional file 1: Table S1.

## Additional file

10.1186/s12867-015-0051-7 Contains raw data (C_q_ values of reference and target genes) of quantitative RT-PCR analysis.

## Abbreviations

93RS2, SCIT-C8: rat Sertoli cell lines
93RShAR17: with human *AR* transfected 93RS cells
AIS: androgen insensitivity syndrome
*Amh*: anti-muellerian hormone
AR/*Ar*: androgen receptor
ARE: androgen responsive element
*Bambi*: BMP and activin membrane-bound inhibitor
*Ccl5*: chemokine (C–C motif) ligand 5
*Ckdn1a*: cyclin-dependent kinase inhibitor 1a
CDS: coding DNA sequence
*Col4a1*: collagen type IV alpha 1
*Cox2*: cyclooxygenase 2
*Cybrd1*: cytochrome b reductase 1
DBD: DNA binding domain
*Dhh*: desert hedgehog
DHT: dihydrotestosterone
*Egr1*: early growth response 1
FBS: fetal bovine serum
FC: fold change
FDR: false discovery rate
*Fgfr2*: fibroblast growth factor receptor 2
*Fst*: follistatin
*Gja1*: gap junction protein alpha 1
GO: gene ontology
IF: immunofluorescence
*Ifnb1*: interferon beta 1, fibroblast
*Ifr7*: interferon regulatory factor 7
*Il*-*1β*: interleukin 1β
*Inhbb*: inhibin beta B
IPA: Ingenuity^®^ Pathway Analyzer
ITS: insulin-transferrin-selenin
TJ: tight junctions
LBD: ligand binding domain
*Myc*: myelocytomastosis oncogene
NTC: no template control
PAGE: polyacrylamide gel electrophoresis
PBS: phosphate buffered saline
*Pmepa1*: prostate transmembrane protein, androgen induced 1
PPCA: probabilistic principal component analysis
*Ptsg2*: prostaglandin-endoperoxide synthase 2
*Rarg*: retinoid acid receptor, gamma
*Rplp*: ribosomal protein l 16
RT-PCR: reverse transcription polymerase chain reaction
RT-qPCR: quantitative RT-PCR
SK-11: WL3 mouse Sertoli cell lines
*Smad 1*: SMAD familiy member 1
*Sox9*: *Sry*-box 9
*Sry*: sex determining reagion on Y chromosome
*Steap2*: six-transmembrane epithelial antigen of the prostate 2
T: testosterone
TBST: Tris-Buffered Saline and Tween 20
*Tf*: transferrin
*Tgfb1i1*: transforming growth factor beta 1 induced transcript 1
*Tnfrsf1a*: tumor necrosis factor receptor superfamily, member 1a
*Ubc*: ubiquitin c
